# Intervention of next-generation sequencing in diagnosis of Alzheimer’s disease: challenges and future prospects

**DOI:** 10.1590/1980-5764-DN-2022-0025

**Published:** 2023-08-07

**Authors:** Tijimol Chandy

**Affiliations:** 1 MedGenome Labs Pvt. Ltd., Bangalore-560100, Karnataka, India.

**Keywords:** Neurodegenerative Diseases, High-Throughput Nucleotide Sequencing, Alzheimer Disease, Exome Sequencing, Whole Genome Sequencing, Doenças Neurodegenerativas, Sequenciamento de Nucleotídeos em Larga Escala, Doença de Alzheimer, Sequenciamento do Exoma, Sequenciamento Completo do Genoma

## Abstract

Clinical diagnosis of several neurodegenerative disorders based on clinical phenotype is challenging due to its heterogeneous nature and overlapping disease manifestations. Therefore, the identification of underlying genetic mechanisms is of paramount importance for better diagnosis and therapeutic regimens. With the emergence of next-generation sequencing, it becomes easier to identify all gene variants in the genome simultaneously, with a system-wide and unbiased approach. Presently various bioinformatics databases are maintained on discovered gene variants and phenotypic indications are available online. Since individuals are unique in their genome, evaluation based on their genetic makeup helps evolve the diagnosis, counselling, and treatment process at the personal level. This article aims to briefly summarize the utilization of next-generation sequencing in deciphering the genetic causes of Alzheimer’s disease and address the limitations of whole genome and exome sequencing.

## RESUMO

O diagnóstico clínico de vários distúrbios neurodegenerativos com base no fenótipo clínico é difícil devido à sua natureza heterogênea e às manifestações da doença que se sobrepõem. Portanto, a identificação dos mecanismos genéticos subjacentes é de suma importância para um melhor diagnóstico e regimes terapêuticos. Com o surgimento do sequenciamento de próxima geração, o diagnóstico se tornou mais acessível com uma abordagem imparcial em todo o sistema para identificar simultaneamente todas as variantes de genes no genoma. Atualmente, vários bancos de dados de bioinformática sobre variantes genéticas descobertas e indicações fenotípicas estão disponíveis online. Uma vez que os indivíduos são únicos em seu genoma, a avaliação com base em sua composição genética ajudou na evolução do processo de diagnóstico, aconselhamento e tratamento em nível pessoal. Este artigo teve como objetivo resumir brevemente a utilização do sequenciamento de próxima geração para decifrar as causas genéticas da doença de Alzheimer (DA) e abordar as limitações do sequenciamento completo do genoma e do exoma.

## NEXT-GENERATION SEQUENCING

Sequencing methods, such as Maxam-Gilbert’s chemical degradation method, were first employed to get fragments sequencing, but were soon replaced by the first-generation sequencing techniques such as Sanger’s chain-termination method, due to its hazardous chemical use and toxicity. Sanger sequencing was the popular rapid method used in the late 1900s for the Human Genome Project (HGP) and was also employed to deduce the gene variants responsible for causing disorders^1,2^. These techniques were efficient, but candidate gene selection and sequencing costs were challenging and time-consuming.

By the early 2000s, high-throughput next-generation sequencing (NGS) technologies were developed, making diagnosis easier and hassle-free. NGS is a combination of biology, statistics and information technology that allows massive parallel sequencing of genomes within a relatively short period of time. It achieves tremendous success in microbial genetics, monogenic diseases and complex diseases such as cancer genomics and other multifactorial syndromes. Recently, neurology also adopted the NGS techniques along with other imaging and biochemical methods to gain more expertise in identifying the variants causing disorders^3^. NGS can also be used to study deoxyribonucleic acid (DNA) methylation, protein DNA interaction, ribonucleic acid (RNA) study (RNA-Seq) etc.^4^.

There are three NGS approaches currently employed namely whole-genome sequencing (WGS), whole-exome sequencing (WES), and gene-targeted panels.

## WHOLE GENOME SEQUENCING

WGS sequences the whole genome together. It helps to uncover variation in any part of the human genome, including coding, noncoding, and mitochondrial DNA (mtDNA) regions. WGS is considered the best option once DNA variations outside protein-coding regions can affect gene activity and protein production, potentially leading to genetic disorders^4^. It also helps to gather more information on an unknown or partially-known disorder and to discover the genomic instabilities leading to complex disorders^5^. It becomes easier to predict any specific variation running in the linage or genetic pool leading to specific phenotypes through various genome-wide association studies (GWAS). In the Encyclopedia of DNA Elements (ENCODE) project, one can see that not only coding regions but also non-coding regions are responsible for causing different complex traits^6^. WGS allows for the detection of copy number variations (CNVs), gross chromosomal abnormalities, and intergenic, regulatory and deep intronic variants, leading to a higher diagnostic yield. In the neurogenetics field, the WGS was first successfully used for the identification of a causative coding mutation in an autosomal recessive neurodegenerative Charcot-Marie Tooth disease^7^. Whole-genome methylation-specific studies can provide important information on how epigenetic and environmental factors alter gene expression.

## WHOLE EXOME SEQUENCING

WES sequences only the exons or protein-coding parts of genes. It is seen that most known disease-causing mutations (∼85%) occur in exons of the gene, hence WES is widely used among clinicians and academics. It is targeted only to exons; therefore, considered a cost-effective method that demands less storage volume (∼4–5 Gb per exome) and reduced time consumption for analysis^8^. WES offers comprehensive coverage and increased sequencing depth which helps in identifying single nucleotide variants (SNVs) and small insertions/deletions (indels) for population genetics, genetic disease research, and cancer studies. It provides a better platform for detecting mutations running in a family using trio analysis which enables couples to plan their family in a better and healthier way. Through the exome enrichment strategy, we can get a more precise view of gene regulation which includes untranslated regions (UTRs) and microRNAs (miRNA). With WES, there are chances of incidental findings, which can give a valuable insight to the existing knowledge of the disease condition and its pathogenesis in various disorders^9^. It helps modify disease diagnosis steps and treatment strategies better.

Gene-targeted panels or custom panels sequence only a few genes that are particularly linked to a specific disorder. Gene-targeted panels are observed to be highly effective in the diagnosis of genetic diseases. It is often very small (250 Kb to 5 Mb) in size thus bringing down sequencing requirements and helping in answering distinct scientific questions quickly. It is an economic and suitable application for finding a particular disease or disorder. However, this approach is limited when it comes to complex neurodegenerative disorders.

## NEXT-GENERATION SEQUENCING WORKFLOW

There are different techniques and pipelines used in sequencing genomes, depending upon the demands at a specific time. But all the methods notably follow three steps in NGS i.e., library preparation, sequencing, and data analysis. The DNA/RNA is extracted first from the tissue sample, then a quality control (QC) check is done to ensure its purity and quantity by ultraviolet (UV) spectrophotometer and fluorometric methods^10^.

Template preparation is the prime step in NGS workflow, where the DNA/complementary DNA (cDNA) library is prepared by fragmenting into numerous small coting by physical, enzymatic, and chemical methods, and attaching adaptors to both ends. These libraries are then amplified either by emulsion PCR (ePCR) in ion torrent sequencing or cluster formation by bridge PCR (bPCR) in Illumina sequencing in different customized sizes and prepared for sequencing. The sequenced library can be directly used for whole-genome analysis or undergo a targeted enrichment process for whole-exome analysis and targeted gene panel testing^11^.

Most clinical sequencing is performed on different types of instruments such as Illumina sequencers including the HiSeq, MiSeq, NexSeq, Pacific Biosciences, Ion Torrent series of machines including the IonPGM, IonProton, and IonS5, and others^12^. The data generated after sequencing is analyzed using different pipelines and software packages. The results obtained will be interpreted based on the requirement of analysis using various sets of bioinformatics tools.

## ALZHEIMER’S DISEASE

Neurodegenerative disorder (NDD), as the name suggests, is a disorder in which cells of the central nervous system stop working or die. They are classified and diagnosed based on clinical features such as physical signs, symptom-onset, and disease course. Alzheimer’s disease (AD) is one of the most common NDDs characterized by dementia that typically begins with subtle mild cognitive impairment (MCI), gradually becomes severe and, finally, leads to total impairment of mental functions. It is commonly seen in the aging population and is becoming a significant cause of socio-economic burden worldwide. Neuropathologic findings mainly extracellular β-amyloid plaques and intraneuronal neurofibrillary tangles (containing tau protein that accumulate in vulnerable brain regions) are the hallmark of AD^13^. Initially, damage occurs in the hippocampus and the entorhinal cortex (memory-forming part of the brain). It then leads to seizure of neuronal function and loose connections of neurons, and gradually to shrinkage of brain parts.

As of 2021, more than 50 million people were affected by dementia worldwide, and this number is estimated to triple to 152 million by 2050 as the world’s population ages^14^. From 1990 to 2019, the incidence and prevalence of AD and other dementias increased 147.95% and 160.84%, respectively^15^.

Four subtypes are identified in AD so far. Familial or Early-Onset Alzheimer’s Disease (EOAD) constitutes less than 2% of total AD; neurological and depressive behaviors are early symptoms of EOAD^16^. Mutations in amyloid precursor protein (APP), presenin 1 (*PSEN1*), and presenin 2 (*PSEN2*), discovered through linkage studies, are the genes predominantly responsible for causing EOAD (Table 1). EOAD is referred to as “Mendelian AD” due to the almost complete penetrance and mostly autosomal-dominant mode of transmission of implicated DNA sequence changes^17^.

**Table 1:** Common genes in early onset Alzheimer’s disease.

Genes	Function of protein*	Pathway of disease	Inheritance	Chromosomal location†
PSEN1	Subunit of gamma-γ-secretase complex; integral membrane protein processing	AβPP Metabolism	AD	14q24.2
PSEN2	Subunit of gamma-γ-secretase complex; integral membrane protein processing	AβPP Metabolism	AD	1q42.13
APP	Transmembrane protein; neural growth and repair	AβPP Metabolism	AD	21q21.3

Abbreviations: PSEN1: presenin 1; PSEN2: presenin 2; APP: amyloid precursor protein; AD: autosomal dominant; AβPP: amyloid-β protein precursor.

* https://www.genecards.org/

† https://omim.org/entry/

With the advent of NGS, clinicians can better trace diseases at molecular level. AD and its associated genes have been researched extensively^18–22^. In the late 1900s and early 2000s, many genes were found to cause Alzheimer’s phenotype through GWAS. In 2003, the first GWAS were initiated in AD and, in 2007, it was published a meta-analysis of AD susceptibility genes. An AD database was then created, called AlzGene (http://www.alzgene.org)^23^.

An estimated 52 pathogenic mutations are identified in the APP gene; most of them are positioned in the vicinity of the β and γ-secretase cleavage sites (exons 16 and 17). Different mutations in V717I/G/F/L and E693K/Q/G/Del residues of APP make them mutation hotspots in the APP gene.

Mutations in exons 5, 6, 7, and 8 of *the PSEN1* gene account for 70% of all identified mutations. Five different mutations of *PSEN1* residue 143 (I143V/F/N/T/M, encoded by exon 5) are identified, making I143 residue a mutation hotspot^24^. It has been discerned that the *PSEN1* variant (p.Thr291Pro), found in an individual presenting with spastic paraplegia, can later precede dementia onset in *PSEN1*-related familial AD^25^.

Several candidate gene approaches and GWAS have been performed to identify new genes related to AD (Table 2). Late-onset AD (LOAD) is reported to be caused by multiple gene; semantic dementia and conceptual formation deficit progress in LOAD. Around 90–95% of AD cases are attributed to sporadic mutations^26^. AD overlaps with other disease-related pathways such as Parkinson’s disease (PD), amyotrophic lateral sclerosis (ALS), frontotemporal dementia (FTD), Huntington disease (HD)^27^.

**Table 2:** Common genes in late onset Alzheimer’s disease.

Genes	Function of protein*	Pathway of disease	Cause	Chromosomal location†
APOE	Redistribution of lipids	Cholesterol metabolism	SNP	19q13.2
CR1	Receptor of complement C3b protein that binds Aβ, mediates innate immunity	Immune response	SNP	1q32.2
CLU	Apoptosis and clearance of cellular debris, lipid transport and inflammation	Cholesterol, immune metabolism	SNP	8p21.1
ABCA7	Transportation of phospholipids and phagocytosis	Cholesterol metabolism	SNP/haplodeficiency	19p13.3
PICALM	Synaptic neurotransmitter release and intracellular trafficking	Endocytosis	SNP	11q14.2
TREM2	Inflammatory response		Loss of function (missense) mutation	6p21.1
SORL1	Vessel trafficking and cargo sorting	Endocytosis	SNP/nonsense and missense mutation; somatic mutations	11q24.1
ADAM10	Mediates integral membrane protein cleavage	AβPP Metabolism	Mutations	15q21.3
BIN1	Endocytosis, inflammation, calcium homeostasis and apoptosis	Tau Pathology	Mutations	2q14.3

Abbreviations: APOE: apolipoprotein E; SNP: single nucleotide polymorphism.

* https://www.genecards.org/

† https://omim.org/entry/

Apolipoprotein E (*APOE*) e4 allele on chromosome 19, identified using Sanger and family-based approaches, significantly contributes to AD diagnosis in homozygous (*APOE* e4/e4) and heterozygous (*APOE* e3/e4) conditions. APOE e4 alleles are strongly associated with AD risk and contribute to various functional abnormalities, neurotoxicity, mitochondrial dysfunction, and cerebrovascular defects^28^.

Various studies have been conducted targeting *ABCA7, BIN1, CLU, CR1, MS4A6A, EPHA1, CD2AP,* and *PICALM* in different genetic pools. Few pathogenic mutations such as splice site, stop mutation, and frameshift deletions were identified suggesting a loss-of-function mechanism associated with LOAD^29^. Several missense mutations were found, of which most variants were classified as of uncertain significance due to the lack of functional studies.

A well-known mutation in *TREM2* [R47H], identified as causing partial loss of function, contributes to Aβ accumulation by attenuating microglial-mediated Aβ clearance^30^. The clinical phenotype of mutations in FTD genes, including *GRID2IP, WDR76, GRN, MAPT*, and *C9ORF72*, can be clinically indistinguishable from typical AD^31^. Rare variants in the *MAPT* gene were found to be associated with AD in patients without ApoE e4 and tau pathology^32^. Loss-of-function or null variants in the *SORL1* gene is a significant genetic risk factor for AD, as the truncated protein may result in disruption of its ability to bind APP^33^.

Homozygous and compound heterozygous *VWA2* mutations mimic autosomal recessive inheritance in sporadic AD cases^34^. A missense variant p.Asp238Glu in *UNC13B* showed segregation within two families of Puerto Rican ancestry and was overrepresented in the AD cases^35^.

A family-based study showed a genome-wide significant linkage peak in 9p21 which overlapped with an AD linkage region. Novel genome-wide significant (GWS) AD-associated non-synonymous variants were identified, as well as a protective variant in *PLCG2* (p.P522R), a risk variant in *ABI3* (p.S209F), and a novel variant in *TREM2* (p.R62H). These genes are highly expressed in microglia and highlight an immune-related protein-protein interaction network enriched for previously identified AD risk genes^36^.

Familial segregation in *PLD3* (V232M) was seen, suggesting that *PLD3* influences APP metabolism, such that overexpression leads to lower Aβ levels while knock-down of *PLD3* leads to increased levels of Aβ^37^. The *MUC6* VNTR repeat expansion influences *AP2A2* gene expression involved in clathrin-coated vesicle function and is associated with AD pathogenesis, particularly tau proteinopathy^38^.

The GGC repeat expansion of *NOTCH2NLC* gene leads to neuronal intranuclear inclusion disease (NIID) and was also observed in family members affected by AD and Parkinson’s disease^39^. A rare nonsynonymous variant in the *SHARPIN* gene, p.Gly186Arg, is potentially associated with increased risk of LOAD. It leads to aberrant cellular localization of the variant protein and attenuates the activation of NF-κB, a central mediator of inflammatory and immune responses^40^.

Individuals with Down syndrome (trisomy 21) developed the AD neuropathologic hallmarks after the age of 40 years, due to overexpression of *APP* on chromosome 21 and the resultant overproduction of β-amyloid in the brains of people’s trisomy for this gene^41^.

Somatic (non-inherited) mtDNA mutations and mitochondrial dysfunction are thought to be important drivers of ageing and age-related neurodegenerative diseases such as AD^42^. The sequencing of *OGDH, DLST*, and *DLD* genes, encoding alpha-ketoglutarate dehydrogenase complex (αKGDHc) subunits, identified a likely pathogenic [R263H] mutation in the *DLD* gene associated with AD^43^.

Few gender-based studies have been conducted on AD disorder; nevertheless, it was found that females are at higher risk. *APOE* e4 females may show increased levels of AD pathology, more compromised brain network integrity, and/or accelerated longitudinal decline at a given level of AD pathology than males^44^. Greater hippocampal electroencephalograph disruption and memory impairment were seen in female *ACE1* [R1279Q] KI mice, compared to males, suggesting a mechanism for higher AD risk in women^45^.

**Figure 1:**
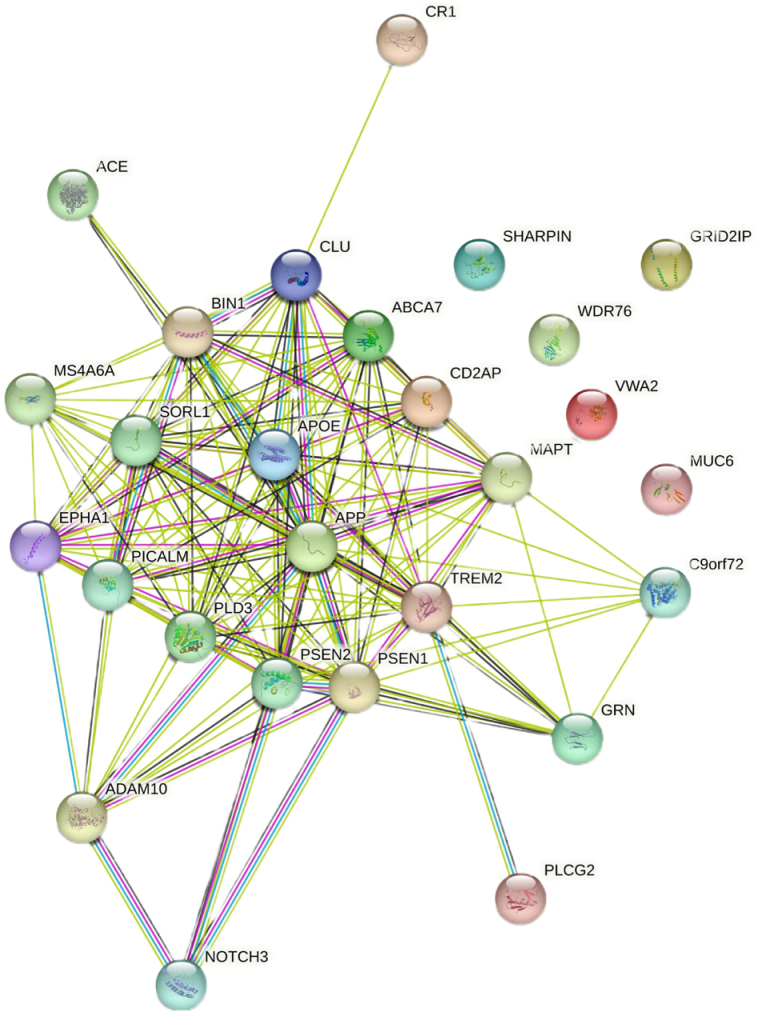
Interaction between the genes associated with Alzheimer’s disease.

Despite these recent advances in AD genomics, a significant part of the genetic contribution to AD remains unexplained. Further functional studies are required to examine mutation-specific expressions and understand the mechanisms by which the mutations lead to disease^46^. Figure 1 presents the interaction between the genes associated with Alzheimer’s disease. The functional links between these genes are identified and documented by experimental, biochemical, and expressional studies in scientific literature.

## CHALLENGES OF WHOLE-EXOME SEQUENCING AND WHOLE-GENOME SEQUENCING

The high complexity of NGS workflow and result interpretation are the major challenges encountered in WES and WGS. Most variants are inevitably detected in every individual tested and it is essential to provide a comprehensive clinical interpretation for these variants with a long time invested^47^. This highlights the fact that the cost for providing clinical WGS/WES is likely to remain high even as sequencing costs fall. Every step of the NGS assay requires thorough validation, therefore the sample undergoes quality checks under standard guidelines. Sequencing errors such as low depth, low alternate allele frequency, low coverage region, etc. occurring due to technical limitations, may lead to a missed variant or a false-positive result.

In genome analysis, variant calling is affected by many factors. First, polymorphic region — a region with multiple variants is scattered throughout the region and is known as a “confetti effect”. Any variant calling in this region can be challenging. Second, homopolymer repeat regions — tracts of repeated small nucleotide sequences together, which are skipped or cannot be picked up by sequencing. Third, strand bias — it occurs when reads aligned to a reference are biased towards the forward or reverse strands. It is common around exon boundaries, particularly for WES with a high chance of a false positive variant or a missed variant^48^. Fourth, low depth of coverage — it means that the number of reads covering a region is few. Sometimes, false calls can be made by assuming polymorphic variant as a rare significant variant^49^. In this case, Sanger sequencing can be used to validate the variant as it tends to provide qualitative results, differently from NGS. Sanger sequencing is ideal for sequencing homogeneous samples that include one template, one gene, or one region.

Most genes referred above have 100% coverage in NGS sequencing. Few genes, such as *PICALM, CD2AP* and *ADAM10*, and *CR1*, are not fully covered due to segmental duplication site (pseudogenes) of polymorphic low covered regions, present in the genes (Table 3)^50^.

**Table 3:** Average gene coverage and sequencing depth expected in sequencing for Alzheimer’s disease genes.

Genes	Average coverage (%)	Average depth
APP	100	105.73
PSEN1	98.89	111.32
PSEN2	100	110.2
APOE	100	92.4
BIN1	100	121.66
CR1	62.73	70.58
CLU	100	119.45
ABCA7	100	117.71
PICALM	93.12	83.1
TREM2	100	123.81
SORL1	99.88	101.53
ADAM10	96.01	83.47
CD2AP	93.58	80.23
MS4A6A	99.74	101.22
EPHA1	100	123.04
GRID2IP	99.79	113.99
GRN	100	139.3
MAPT	100	185.73
C9ORF72	96.79	76.9
NOTCH3	99.77	151.88
PLCG2	99.86	127.72
PLD3	100	113.51
ACE	99.91	112.06
WDR76	99.87	106.15
VWA2	100	127.97
MUC6	100	374.11
SHARPIN	100	132.06

Abbreviations: APP: Amyloid Beta A4 Precursor Protein; PSEN1: Presenilin 1; PSEN2: Presenilin 2; APOE: Apolipoprotein E; BIN1: Bridging Integrator 1; CR1: Complement Component Receptor 1; CLU: Clusterin; ABCA7: ATP-Binding Cassette, Subfamily A, Member 7; PICALM: Phosphatidylinositol-Binding Clathrin Assembly Protein; TREM2: Triggering Receptor Expressed on Myeloid Cells 2; SORL1: Sortilin-Related Receptor; ADAM10: A Disintegrin And Metalloproteinase Domain 10; CD2AP: CD2-Associated Protein; MS4A6A: Membrane-Spanning 4-Domains, Subfamily A, Member 6A; EPHA1: Ephrin Receptor EphA1; GRID2IP: GRID2-Interacting Protein 1; GRN: Granulin Precursor; MAPT: Microtubule-Associated Protein TAU; C9ORF72: Chromosome 9 Open Reading Frame 72; NOTCH3: Notch Receptor 3; PLCG2: Phospholipase C, Gamma-2; PLD3: Phospholipase D Family, Member 3; ACE: Angiotensin I-Converting Enzyme; WDR76: WD Repeat-Containing Protein 76; VWA2: Von Willebrand Factor A Domain-Containing Protein 2; MUC6: Mucin 6, Gastric; SHARPIN: Shank-Associated RH Domain Interactor.

In a targeted-panel sequencing, the clinical importance of the genes and selective enrichment of targeted-genomic areas for NGS are the primary concerns. The selection of suitable target capture approaches and sequencing methods are crucial in yielding good quality results. This is determined by several factors such as the sample type (fresh, frozen, or formalin-fixed paraffin-embedded [FFPE]), quantity and quality of DNA or RNA routinely available^51^.

As it is evident that every variation cannot be classified as pathogenic, a thorough validation is required before sending a final report to the patient. Research is a continuous process and functional studies on different variations can lead to upgrading or downgrading a variant classification. A revision of the variant in reports must be done timely so that clinicians can design and provide adequate treatment to patients.

Failure to state the authenticity of large deletions or duplications in genes (copy number variations) can lead to serious disorders. Therefore, cross-confirmatory tests are recommended to ascertain the CNV and its effect on the patient’s phenotype, so that treatments can be planned accordingly^52^.

In conclusion, over the past couple of decades, high-throughput genome technologies have changed the genetic landscape of AD. NGS combined with other molecular advances, such as omics data, biochemical and functional studies, can now provide scientists with the ability to gain a comprehensive view of molecular disease pathways.

NGS assists in deducing the gain or loss of function in genes responsible for causing AD. Moreover, recent advances in NGS and its analysis have helped detect and confirm short tandem deletions and duplications. Several modifications to the current technology have been made on a day-to-day basis so that people can yield maximum benefit from NGS and help them lead a better and quality life. In the near future, both hypothesis-free (whole-genome, whole-exome) and hypothesis-driven (targeted-exome) NGS approaches will probably disentangle much of the disease genetics. Despite the few limitations stated above, it can pave the way for developing novel therapeutics and designing the right treatment on an individual level, called personalized medicine or precision medicine, which can effectively prevent or halt the progression of this devastating disease.
